# Genetical control of 2D pattern and depth of the primordial furrow that prefigures 3D shape of the rhinoceros beetle horn

**DOI:** 10.1038/s41598-020-75709-y

**Published:** 2020-10-29

**Authors:** Haruhiko Adachi, Keisuke Matsuda, Teruyuki Niimi, Shigeru Kondo, Hiroki Gotoh

**Affiliations:** 1 Graduate School of Frontier Biosciences, Osaka University, Suita, Osaka 565-0871 Japan; 2 Osaka University Hospital, Osaka University, Suita, Osaka 565-0871 Japan; 3 Division of Evolutionary Developmental Biology, National Institute for Basic Biology, Okazaki, Aichi 444-8585 Japan; 4 Ecological Genetics Laboratory, Department of Genomics and Evolutionary Biology, National Institute of Genetics, Mishima, Shizuoka, 411-8540 Japan

**Keywords:** Developmental biology, Genetics

## Abstract

The head horn of the Asian rhinoceros beetle develops as an extensively folded primordium before unfurling into its final 3D shape at the pupal molt. The information of the final 3D structure of the beetle horn is prefigured in the folding pattern of the developing primordium. However, the developmental mechanism underlying epithelial folding of the primordium is unknown. In this study, we addressed this gap in our understanding of the developmental patterning of the 3D horn shape of beetles by focusing on the formation of furrows at the surface of the primordium that become the bifurcated 3D shape of the horn. By gene knockdown analysis via RNAi, we found that knockdown of the gene *Notch* disturbed overall horn primordial furrow depth without affecting the 2D furrow pattern. In contrast, knockdown of *CyclinE* altered 2D horn primordial furrow pattern without affecting furrow depth. Our results show how the depth and 2D pattern of primordial surface furrows are regulated at least partially independently during beetle horn development, and how both can alter the final 3D shape of the horn.

## Introduction

The insect body is covered with a rigid exoskeleton, so growth in body size is achieved via molting. Prior to a molt, the epidermis detaches from the rigid exoskeleton and undergoes a period of rapid growth before laying down a new cuticle inside the old one. Although larger, this new cuticle must still fit within the rigid confines of the older cuticle until the animal tears out of the old cuticle and expands and hardens the new one^1–3^. During the final larval molt of holometabolous insects this feat is especially complicated, since the newly forming cuticle may have a shape that differs markedly from the prior one, including entirely new structures not present in larvae such as wings, genitalia, and secondary sexual structures like horns^4,5^. Packing these new structures within the confined space of a smaller, larval cuticle is accomplished through precise patterns of cuticular furrows, or folds. Although all insects must include some form of furrowing as they molt, and the details of this furrowing process likely relate to the relative amounts of localized growth and the final size and form of adult structures, the mechanisms regulating cuticular furrow formation are largely unexplored.

In male rhinoceros beetles, the exaggerated horn develops beneath the larval cuticle, first appearing during a relatively short period of time (within 2 h) at pupation^6^. The horn primordia consists of a furrowed epithelial cell sheet with soft new cuticle, located under the rigid older cuticle of the larval head capsule. At molting, the primordium is unfurled to form its final 3D shape using fluid pressure from hemolymph, much like blowing up a balloon. Remarkably, this transformation from a folded primordium to a fully extended horn does not require any living cell activities^6^. This indicates that the information for the final 3D structure of the beetle horn is patterned within the primordium by this time.

The horn primordium consists of a mushroom-like macro structure (hereafter “macro structure”) and surface micro furrows (hereafter “micro furrows”)^6,7^ (Supplementary MovieS1, Supplementary Fig. S1). Both the macro structure and micro furrows contribute to the final 3D shape of the beetle horn^6,7^. In our previous study, we showed that anisotropy of cell division contributes to macro structure formation, especially in the stalk region of the mushroom-like structure^6,7^. What remains unknown is the morphological, molecular and cytological properties of the micro furrows, which are not well understood. In this study, we investigated genetic factors regulating the pattern of micro furrowing in the developing beetle head horn primordia, and linked them to the final 3D structure of the horn. We focused on the cap region of the mushroom-like primordia because this region is crucial for the four-pronged horn shape^6,8^. The cap region in a horn primordium has four morphological parameters: the (1) size of macro structure, and the (2) depth, (3) density and (4) 2D pattern (the direction and shape) of the micro furrows. By specifying the precise pattern of cuticular folding, these parameters directly determine the 3D shape of the adult horn. Our objective was to study how these four parameters are regulated. First, we focused on size-dependent differences of the beetle horn to clarify which parameters are dependent on body size and which are not. Second, we screened genes that produced RNAi-induced abnormal head horn phenotypes to find genes which affect the primordial parameter(s) accounting for final horn shape.

## Results and discussion

### The depth of the furrow and 2D furrow pattern are independent of body size

**Figure 1:**
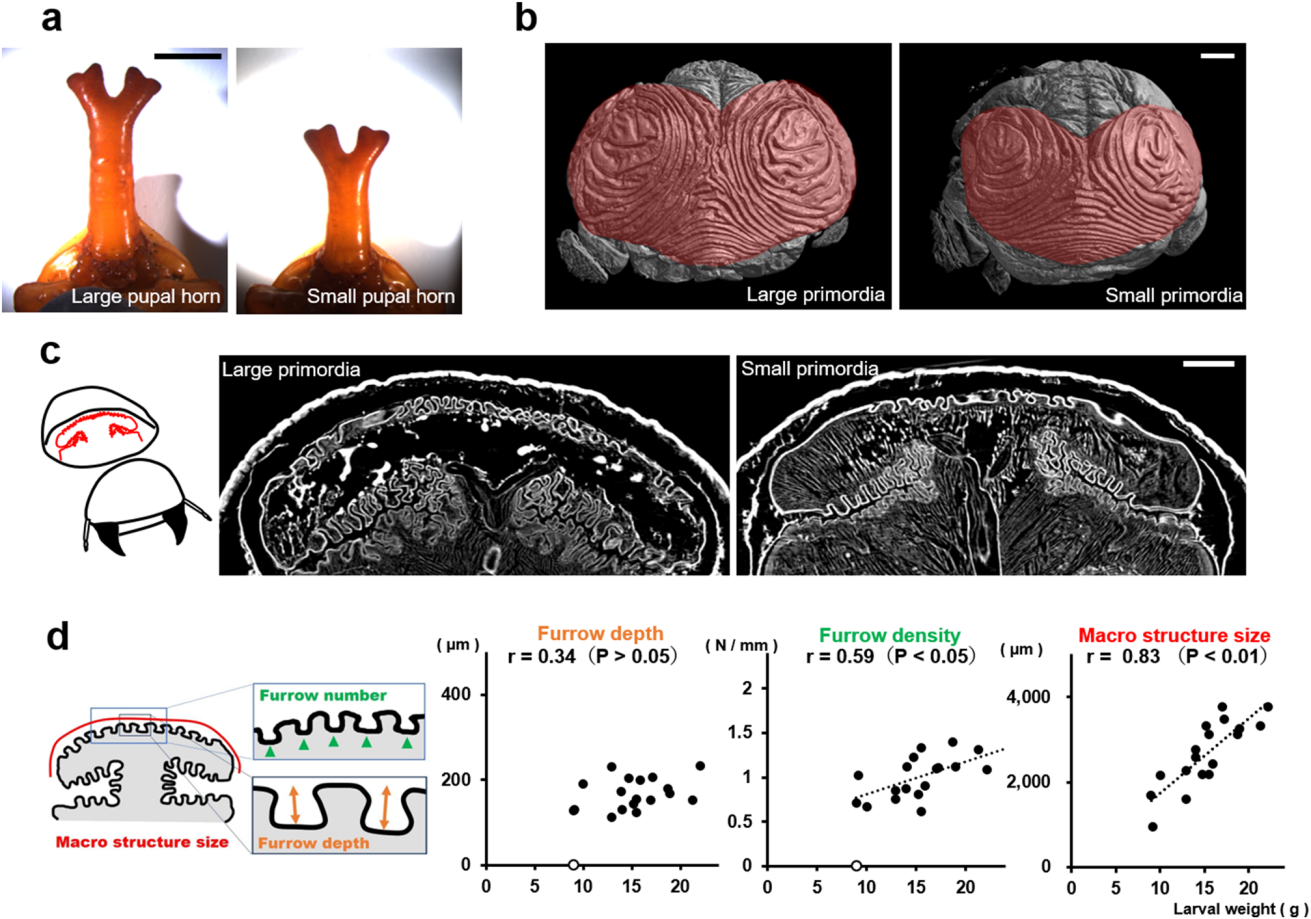
Relationship between body size and the morphology of the primordia. **(a)** The horns of pupae with different body sizes (left: 18 g, right: 14 g). The larger beetle has the longer horn. **(b)** The primordia of the horn from different body sizes (left: 18 g, right: 14 g). Both of the primordia have similar 2D furrow patterns (concentric furrow patterns and stripe furrows between them), while the overall sizes of primordia are different. The cap (top) regions of the primordia are indicated in red. The images were acquired by µCT scanning. **(c)** Virtual frontal section images of horn primordia via µCT scanning. The mushroom-like macro structure can be recognized regardless of body size. **(d)** Relationship between body size and the macro structure size, the density of the furrows, and the depth of the furrows. For macro structure size the correlation coefficient was 0.83 (p < 0.01), for furrow density the correlation coefficient was 0.59 (p < 0.05), and for furrow depth, the correlation coefficient was not significant. In the smallest beetle (white plot: 8.95 g), obvious furrows could not be detected so it was excluded from the analysis (n = 19). Scale bar is 10 mm for **(a)**, 1 mm for **(b)** and **(c)**.

Pupal beetle horns are known to differ in length depending on body size, mainly due to growth conditions^8–12^ (Fig. 1a). The size of the four-pronged tip region of the horn also covaried with body size (Supplementary Fig. S2). We investigated how the macro structure and micro furrows of the horn primordia contributed to variation in the size of the tip region and whether these parameters fluctuated simultaneously or independently. We analyzed the depth and density of furrows and mushroom-like macro structure size in different sized primordia because these factors can change the horn size (Supplementary Fig. S3). We found that the depth of furrows was constant regardless of the body size (Fig. 1c,d). The density of furrows was negatively correlated with body size, but its correlation coefficient was moderate (Fig. 1c,d) or did not correlate in some areas (Supplementary Fig. S4 and S5). On the other hand, the macro structure size of the primordium was strongly correlated with body size (Fig. 1c,d, Supplementary Fig. S4). This tendency (constant furrow depth, weak or no correlation of furrow density and strong positive correlation of macro structure) was also observed other regions of the horn primordium (Supplementary Fig. S4 and S5).

We also observed primordial surface 2D furrow patterns among different body sized animals. Specifically, we compared the surface furrow pattern (a pair of concentric-like furrows pattern) in both small and large larval primordia (Fig. 1b). These data suggested that size-dependent morphological change in horns is primarily controlled by changing the size of the macro structure, rather than the depth of the primordium furrows.

### The Notch and cyclinE genes contribute to the control of furrow depth and 2D furrow pattern, respectively

Our observations suggested that the depth and 2D patterns of the furrows are strictly regulated independently of body size. Hence, we next searched for the gene(s) involved in controlling furrow depth and 2D pattern. Methodology for use of RNAi is well established for beetles^13,14^, and horn shape can be changed by specific gene knockdown^12,15–18^. We analyzed six candidate genes (*Notch* < *N* > , *CyclinE* < *CycE* > , *dachsous* < *ds* > , *mushroom body defect* < *mud* > , *Optix* < *Optix* > , *Retinal Homeobox* < *rx* >), each already known to affect horn shape^7,15,17^, using RNAi to see how they affected the furrows on the horn primordia.

**Figure 2:**
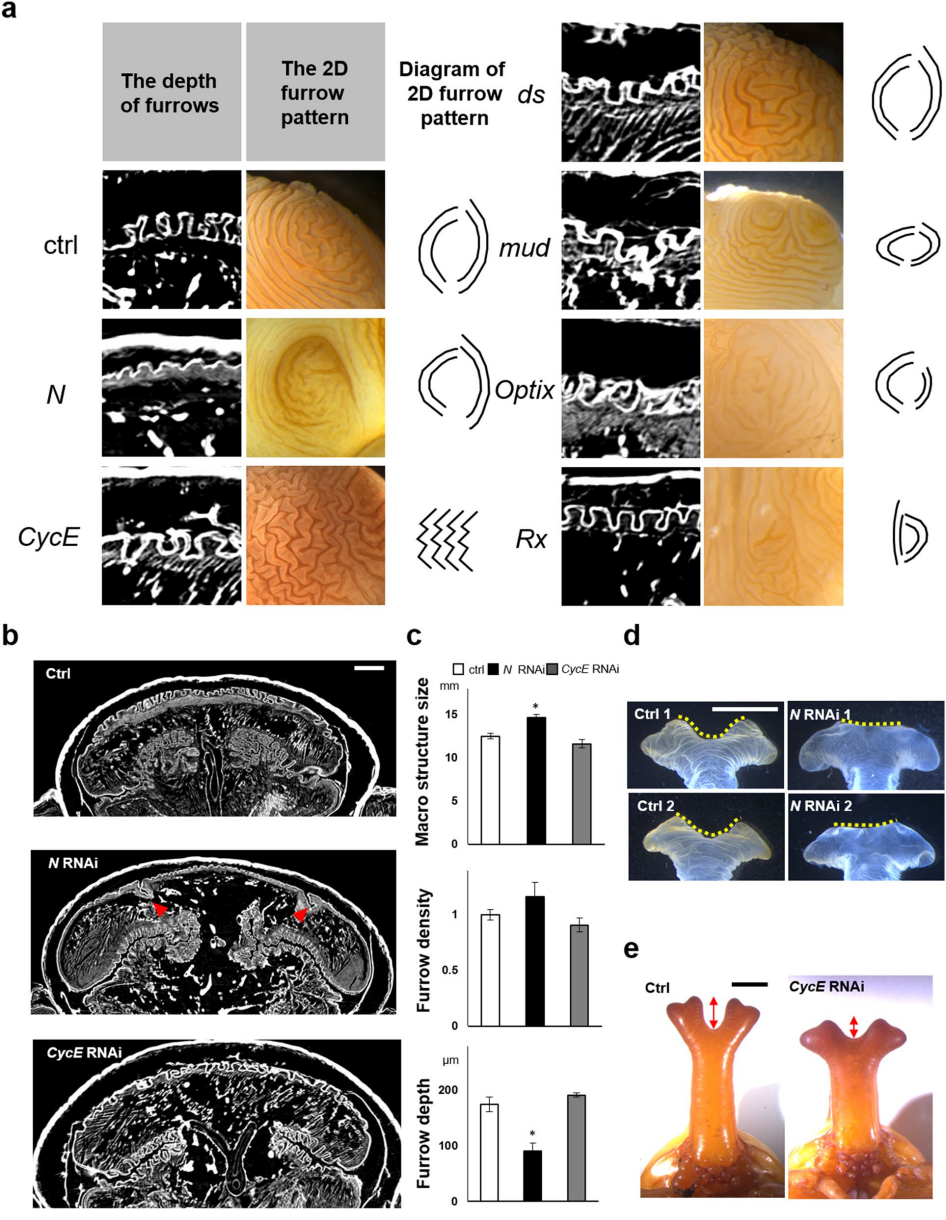
RNAi analysis show that the *Notch* and *CyclinE* genes contribute to control of furrow depth and 2D furrow pattern, respectively. **(a)** Comparison of the depth and surface 2D pattern (concentric-like pattern) of the horn primordia between control and six different RNAi treatments. The 2D furrow patterns inside of the primordial top region were compared. *N* RNAi decreases the depth of the furrow and *CycE* RNAi disturbed the concentric-like 2D furrow pattern. **(b)** Comparison of the frontal section of the head just before pupation in control and *N* RNAi and *CycE* RNAi. The red arrowheads show the specific deeper furrows detected in *N* RNAi. **(c)** Quantitative data of the macro structure size and the density and the depth of the furrow (asterisk means P < 0.05) (n = 7, 5, 6 for negative control, *N* RNAi, *CycE* RNAi, respectively; however the furrows were too shallow to measure in one *N* RNAi so that n = 4 for furrow depth and density). **(d)** Pupal horn shape phenotypes between control and *N* RNAi. Two different individuals were shown in both of control and *N* RNAi. Marked differences of center groove depth were highlighted by yellow dashed line. **(e)** Comparison of the pupal horn shape between control and *CycE* RNAi. Marked differences of center groove depth were highlighted by red arrows. Scale bar indicates 1 mm for **(b)**, 5 mm for **(d)** and **(e)**.

Although we found several differences in primordium development in RNAi animals, we focused especially on *N* RNAi and *CycE* RNAi because these two changed the depth and 2D pattern of furrows (Fig. 2a) more drastically than did the other candidate genes. *N* RNAi significantly decreased the depth of furrows relative to controls (*egfp* RNAi) in almost all measured regions of the primordium (Fig. 2b,c, Supplementary Fig. S7), except for a pair of specific furrows (Fig. 2b red arrowhead). In addition to decreasing furrow depth, macro structure size and the furrow density were increased in *N* RNAi beetles (Fig. 2c, Supplementary Fig. S7). We then manually extended the primordia to investigate the influence of changes in furrow depth on the final horn shape, because none of the *N* RNAi larvae survived until pupation. We found that in these manually extended horns the center groove and side groove were missing, and the shape of the horn tips were similar to the centroclinal primordial shape (Fig. 2d). On the other hand, *CycE* RNAi changed the 2D furrow pattern (from concentric-like to zigzag-like) (Fig. 2a, Supplementary Fig. S8) and the resulting pupal horn shape (the center groove became shallower) (Fig. 2e), while the macro structure size, and the density and depth of the furrows were not affected (Fig. 2b,c, Supplementary Fig. S7).

Recently, the contribution of Notch signaling to horn formation was also demonstrated in a dung beetle, *Onthophagus taurus*^19^. Our result is consistent with this, and we have found that Notch plays an important role in determining the final horn shape via regulating primordial furrow depth. In addition to the depth of the furrows, the density of the furrows and macro structure size of the primordia were also affected by *N* RNAi. Considering that the macro structure size and the density of the furrow can vary depending on body size (Fig. 1c,d), while the furrow depth did not, it is assumed that *N* RNAi has a direct effect on the depth of the furrow (i.e. the furrow density increasing in *N* RNAi likely to be a byproduct of the effect on furrow depth).

As for the relationship between the morphology of the primordium and pupal horn in *N* RNAi, it is suggested that the change of the depth of furrows causes the change of the final horn shape. Also, in *CycE* RNAi, it is presumed that the change of 2D furrow pattern causes the change of the pupal horn shape, because the different 2D furrow patterns can be extended to variable 3D structures^6^. These results strongly indicate that both furrow depth and 2D furrow pattern are important parameters affecting final horn shape, but that they are regulated independently.

### The Notch and CyclinE genes contribute to the control of frequency and localization of mitosis, respectively

**Figure 3:**
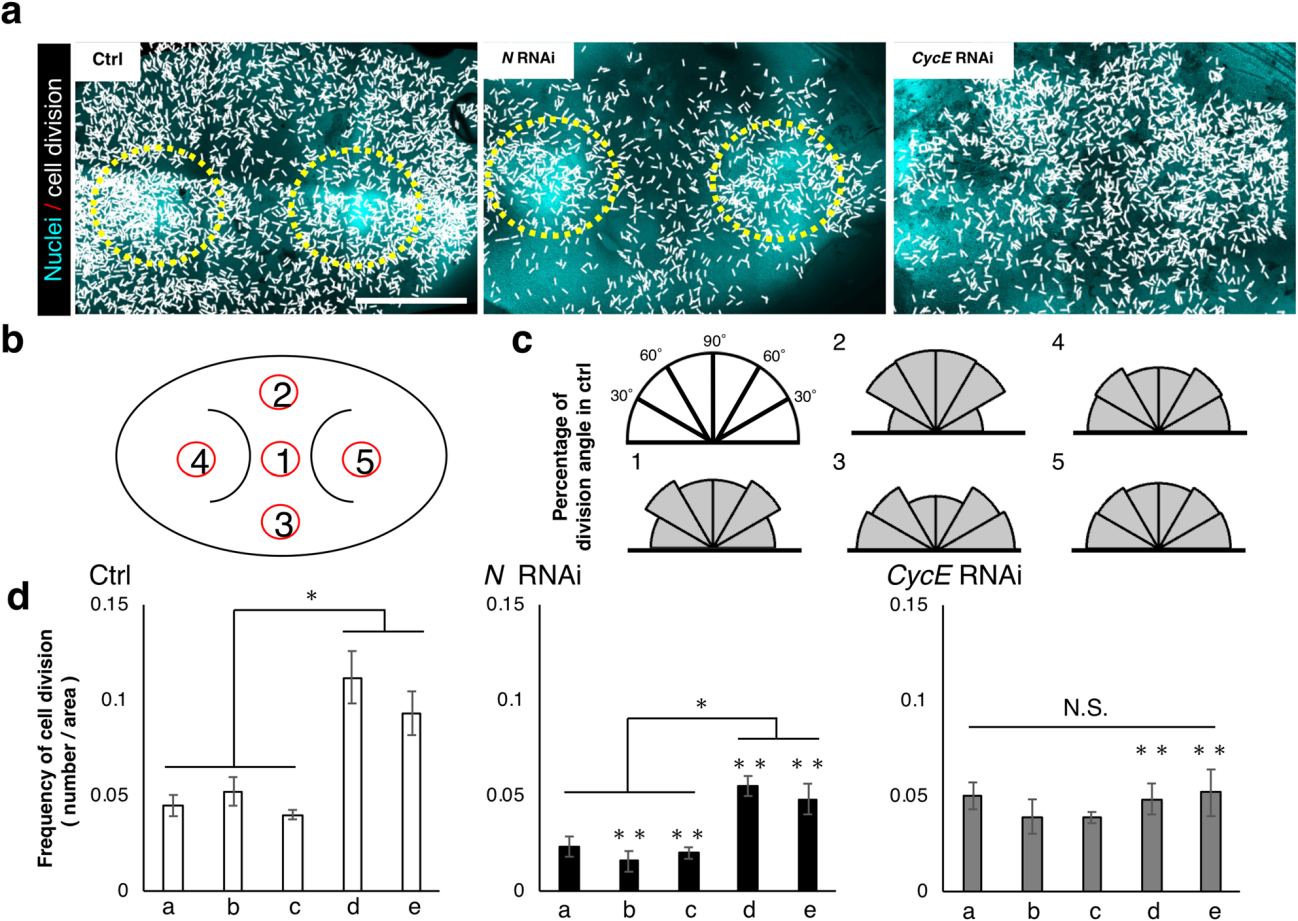
Analysis of cell division of the primordia. **(a)** Plot of the orientation and the localization of cell division in control and *N* RNAi and *CycE* RNAi. Each white line indicates one dividing cell. Direction of white line shows the orientation of cell division at that point. Yellow circles show the area of high intensity of Hoechst fluorescence. **(b)** The area of measured cell division. Five regions were determined by using a pair of characteristic crescent shape furrows as landmarks. This furrow stably formed as the first furrow in all of the analyzed primordia (Supplementary Figure S10). **(c)** Plot of the percentage of the orientation of cell division in five areas of the primordia. Significant change of cell division orientation was not detected (n = 5, for negative control). The results for RNAi are shown in Supplementary Figure S9. **(d)** Quantification of frequency of cell division in five areas of the primordia. In control and *N* RNAi, the frequency of mitosis in the right and left parts were twice as high as in the other parts. *N* RNAi decreased the number of cell divisions in all areas of the primordia. *CycE* RNAi decreased cell division only in both of the side areas of the primordia. Single asterisk (*) indicates significant (P < 0.05) difference between the primordia areas in each RNAi treatment. Double asterisk (**) indicates the statistical significance comparing the same area of primordia between *N* or *CycE* RNAi and negative controls (n = 5, 4, 5 for negative control, *N* RNAi, *CycE* RNAi, respectively). Scale bar indicates 1 mm for **(a)**.

Next, in order to investigate the relationship between cell division and 2D furrow pattern and furrow depth, we analyzed the orientation, frequency and localization of cell division in the mushroom-shaped cap region (Fig. 3a) among control, *N* RNAi and *CycE* RNAi primordia.

In all of the experimental groups, including the control, there was no clear anisotropy of cell division in any measured region of the primordial cap (Fig. 3b,c, Supplementary Fig. S9). Thus, region-specific anisotropy of cell division is not likely to be involved in furrow depth control and region-specific 2D furrow patterns. On the other hand, in control primordia, a specific localization pattern of mitosis was observed among the regions (Fig. 3d). The frequency of mitosis in the right and left parts were twice as high as in the other parts (Fig. 3d). In *N* RNAi, total mitosis was decreased in all of the measured regions, while its distribution pattern was not changed (Fig. 3d). In *CycE* RNAi, the specific localization pattern of cell division was disturbed. That is, the frequency of mitosis in the right and left parts of the primordium was decreased, which resulted in a uniform distribution of mitosis across the regions (Fig. 3d).

A number of studies have reported that Notch contributes to cell proliferation in insect development (i.e. labrum of *Tribolium castaneum*, the eye and the wing of *Drosophila melanogaster*)^20–22^. Hence, in beetle horn development, Notch is also assumed to contribute to cell proliferation. However, the relationship between the frequency of mitosis and the depth of the primordial furrow is still unknown. Notch signaling is also well known to contribute to joint formation in insects^23^. The mechanism of furrow formation may be similar to joint formation because both of them include the folding of epithelial cell sheets^24^. In the future, research about joint formation may provide avenues to further understand furrow formation.

As can be seen from nuclei staining images, the fluorescence intensity of Hoechst was higher in specific areas of control and *N* RNAi horn primordia (Fig. 3a, yellow circle), but this fluorescence pattern was not detected in *CycE* RNAi. This means these brighter areas contained more cells in the S/G2 phase of the cell cycle because the fluorescence intensity of Hoechst is dependent on ploidy. The *CycE* gene is involved in the progression of the cell cycle, especially the transition from G1 to S phase^25^. Hence, it assumed that *CycE* RNAi decreased mitosis in a specific area. The region where the frequency of cell division was disturbed in *CycE* RNAi and the region where the 2D furrow pattern (concentric-like pattern) was disturbed in *CycE* RNAi were identical, suggesting that the cell division distribution pattern can affect the 2D furrow pattern. Although the developmental link between the cell division distribution and final 2D furrow pattern is still unknown, one of the possibilities is that specific mechanical stress caused by differences of cell division frequency may determine the direction of furrows.

## Conclusion

**Figure 4:**
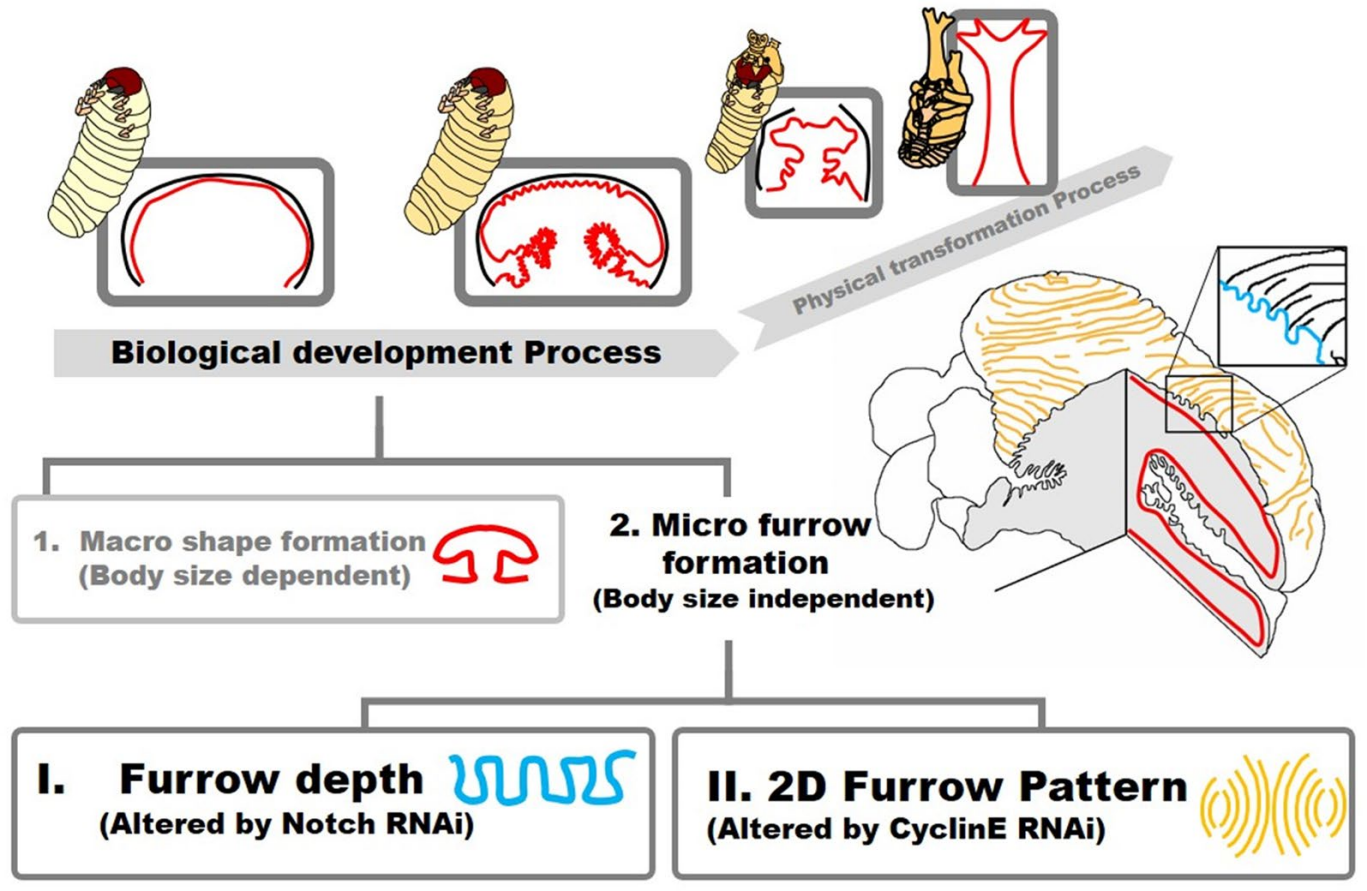
Summary of beetle horn development via “folding and extension”. In the “folding and extension” seen in beetle horn development, the final 3D shape has been already prefigured when the folding process is done. In this study, we demonstrated that the folding process can be divided into (1) macro structure formation and (2) micro furrow formation. Micro furrow formation can be further divided into depth regulation and 2D pattern formation via Notch and CyclinE control, respectively.

In this study, we revealed that morphological parameters (macro structure and micro furrow 2D pattern and depth) in the horn primordia were controlled by different mechanisms. That is, macro structure size is controlled in a body-size dependent manner while micro furrow depth and 2D pattern are not. The depth and 2D pattern of micro furrows are controlled independently via Notch signaling and CyclinE-dependent mechanisms, respectively (Fig. 4). Any one of these morphological parameters can be changed independently, which then alters the final 3D horn shape as well.

## Material and methods

### Insects

The beetle larvae were purchased and kept according to our previous studies^6^. Briefly, commercially purchased last instar (third instar) larvae of the Asian rhinoceros beetle *Trypoxylus dichotomus* were kept individually in 1 L or 800 mL plastic bottles filled with rotting wood flakes at 10–15 °C to suspend their development. Larvae were moved to 25 °C to restart their development before the experiments and/or observations. Bottles were checked daily in order to record the initial date of pupal chamber formation. We defined Day 1 as the first day when the pupal cell was clearly recognized. Most male prepupae pupated at Day 8, therefore we used Day 8 horn primordium with brownish color (a sign of sclerotization) as fully developed ones. Pupae and prepupae were weighed before each experiment. We used 7.28 to 18.05 g pupae and 8.95 to 21.30 g prepupae for detecting size-dependent parameters (Fig. 1). For RNAi gene knockdown and subsequent RNAi-induced horn primordia phenotype analyses, we used a range of pupae from 13.66 to 23.35 g.

### Analysis of pupal horn morphology

Pupal horns were photographed using a digital camera (MZ16FA, Leica, Germany). The inflection point was used as a landmark and the length was measured (Supplementary Fig. S2) using Fiji (Image J). For the length of the side groove and cap side stalk, both sides (right and left) were measured and averaged.

### Degradation of inner structure of the primordia

The primordia have an inner structure containing muscle, tracheae and body fluid in addition to cuticle. To observe cuticular structure clearly, we degraded the inner tissue by incubating overnight with 10% KOH solution at 60 ℃ followed by washing with DDW and dehydration with ethanol. The dehydrated sample was soaked with t-butanol and freeze-dried. The sample was used for µCT observation and expansion experiments to observe the final horn shape of notch RNAi.

### Manual extension of horn primordia

As none of *N* RNAi larvae survived until pupation, we conducted manual extension of the primordia to investigate the pupal horn phenotype. We dissected out fully developed horn primordia from Day 8 prepupae and treated them with 10% KOH to degrade internal tissues. Then, horn primordia were manually extended with forceps. The top region of the primordia, where we mainly focused, was easy to extend. We stably observed the four-pronged horn phenotype in control horns and horns with shallower groove phenotypes resulting from *N* RNAi. On the other hand, it was difficult to fully extend the stalk region of the primordia (cylindric region, determining the length of the horn). Thus, we did not mention the length of the horn due to possibility of artifact.

### µCT scanning

Prepupae were anesthetized on ice and frozen using liquid nitrogen. The frozen samples were truncated by sawtooth. The truncated heads were dried using a freeze-drying system (FZ-2.5, Asahi Life Science, Japan) over 7 h. Then the dried samples were scanned using a micro-CT scanner (Skyscan1172, Bruker, USA) following the manufacture's instruction. The X-ray source ranged from 40 kV, and the datasets were acquired at a resolution of 9 μm/pixel. The stacks of transverse sections were reconstructed from primary shadow images using SkyScan software NRecon (version 1.7.1.0, Bruker, USA). From these image stacks, 3D volume-rendered images were constructed using SkyScan software CT Vox (version 1.5.4.0, Bruker, USA). Skyscan softwares were downloaded from manufacture's website (https://www.bruker.com/products/microtomography/micro-ct-software/3dsuite.html).

### Analysis of the horn primordium morphology

The equal-section images were obtained using landmarks such as the points changing furrow direction and inflection points of macro structures (Supplementary Fig. S6) using SkyScan software DataViewer (version 3.3.0 r1403, Bruker, USA). First, the length of the cap region of the mushroom-like macro structure of the section images was measured. Then the density of furrows was calculated from the number of furrows per the length of measured region. The average depth of individual furrows was calculated from the depth of all furrows in the measured region. All data were measured using Fiji (Image J). Also, in the bottom region of the mushroom-like macro structure, the same analysis was conducted.

### Nuclei staining

Nuclei staining and observation was performed as previous study^7^. Briefly, prepupal heads were fixed PFA for 2 days at 4 ℃ and then horn primordia were dissected under a binocular microscope. The dissected primordia were washed with PBS for three times and incubated at room temperature for 60 min with Hoechst 33342 (1:1000, Invitrogen) in blocking solution (1% BSA). After three PBS washes, primordia were mounted on a glass slide and covered with a glass cover slip. Fluorescent images were observed and recorded with a confocal laser scanning microscope (LSM-780; Carl Zeiss, Jena, Germany).

### Analysis of cell division

Dissected cap area tissue from developing horn primordia at prepupal Day 4 were stained by Hoechst because the timing is just before formation of many micro furrows (Supplementary Fig. S10) and cell division occurs frequently during this time. All of the dividing cells and the angle of each division of the primordia were measured using Fiji (Image J)^26^. The five regions (center, upper, lower, left, and right) were determined based on a pair of crescent-shape furrows.

### Statistical analysis

The morphological parameters, the number and the angle of cell division in Ctrl, *N* RNAi, *CycE* RNAi were conducted in one-way analysis of variance (ANOVA test) followed by the Dunnet test using R console to compare the difference to controls. In this comparison, the number and angle of cell division in each region of *N* RNAi or *CycE* RNAi primordia (1, 2, 3, 4, 5 in Fig. 3b) were compared to the corresponding region of negative control primordia. For comparing the number and the angle of cell division among regions in each RNAi treatment, multiple comparison analyses were carried out using one-way ANOVA followed by Tukey's honest significant difference test.

### Gene knockdown via RNAi

We searched for the ortholog mRNA sequence from the RNAseq database of *T. dichotomus* (PRJDB6456) using *D. melanogaster* sequences as a query via the tblastn program^27^. Amplification of target gene sequence for making template, synthesis of dsRNA and injection for RNAi were performed as described in our previous study^7^. 5 µg of dsRNA of each target gene was injected into late 3rd instar larvae. The primer sequences for amplifying target gene sequence were listed in Supplementary Table S1.

## Supplementary information

Supplementary Information 1.

Supplementary Video 1.

## Data Availability

There are no datasets for this manuscript to share via public repository.
