# Supplementary material for: Genetical control of 2D pattern and depth of the primordial furrow that prefigures 3D shape of the rhinoceros beetle horn
Source: Sci Rep. 2020 Oct 29;10:18687. doi: 10.1038/s41598-020-75709-y (PMC7596553; doi:10.1038/s41598-020-75709-y)
Supplement: Supplementary file 1 — Supplementary Information 1. [file 41598_2020_75709_MOESM1_ESM.pdf]

Genetical control of 2D pattern and depth of the primordial furrow that prefigures 3D shape of the rhinoceros beetle horn

Haruhiko Adachi[1], Keisuke Matsuda[1,2], Teruyuki Niimi[3], Shigeru Kondo[1], Hiroki Gotoh[4]*

1. Graduate School of Frontier Bioscience, Osaka University, Suita, Osaka, 565-0871, Japan

2. Osaka University Hospital, Osaka University, Suita, Osaka, 565-0871, Japan

3. Division of Evolutionary Developmental Biology, National institute for basic biology, Okazaki, Aichi, 444-8585, Japan

4. Ecological Genetics Laboratory, Department of Genomics and Evolutionary Biology, National Institute of Genetics, Mishima, Shizuoka, Japan

*Corresponding author and address. Ecological Genetics Laboratory, Department of Genomics and Evolutionary Biology, National Institute of Genetics, Mishima, Shizuoka, 411-8540, Japan; E-mail: h-r-goto@ees.hokudai.ac.jp

Fig. S1. The morphology of the horn primordia

Fig. S2. Relationship between body size and the morphology of the pupa

Fig. S3. Three ways to increase the size of horn via changing morphological parameters of the primordia

Fig. S4. Relationship between body size and the morphology of the primordia (sagittal section)

Fig. S5. Relationship between body size and the morphology of the primordia (cap bottom region)

Fig. S6. The primordial sectioning point to analyze the morphological parameters

Fig. S7. Morphological parameters of the primordia in *N* and *CycE* RNAi

Fig. S8. Comparison of 2D furrow pattern between control and *N* and *CycE* RNAi

Fig. S9. Comparison of cell division angle between control and *N* and *CycE* RNAi

Fig. S10. Chronological observation of the primordial furrow formation

Table S1: List of primer used for gene amplification as dsRNA synthesis temperate

Supplementary Movie 1 The morphology of the horn primordia

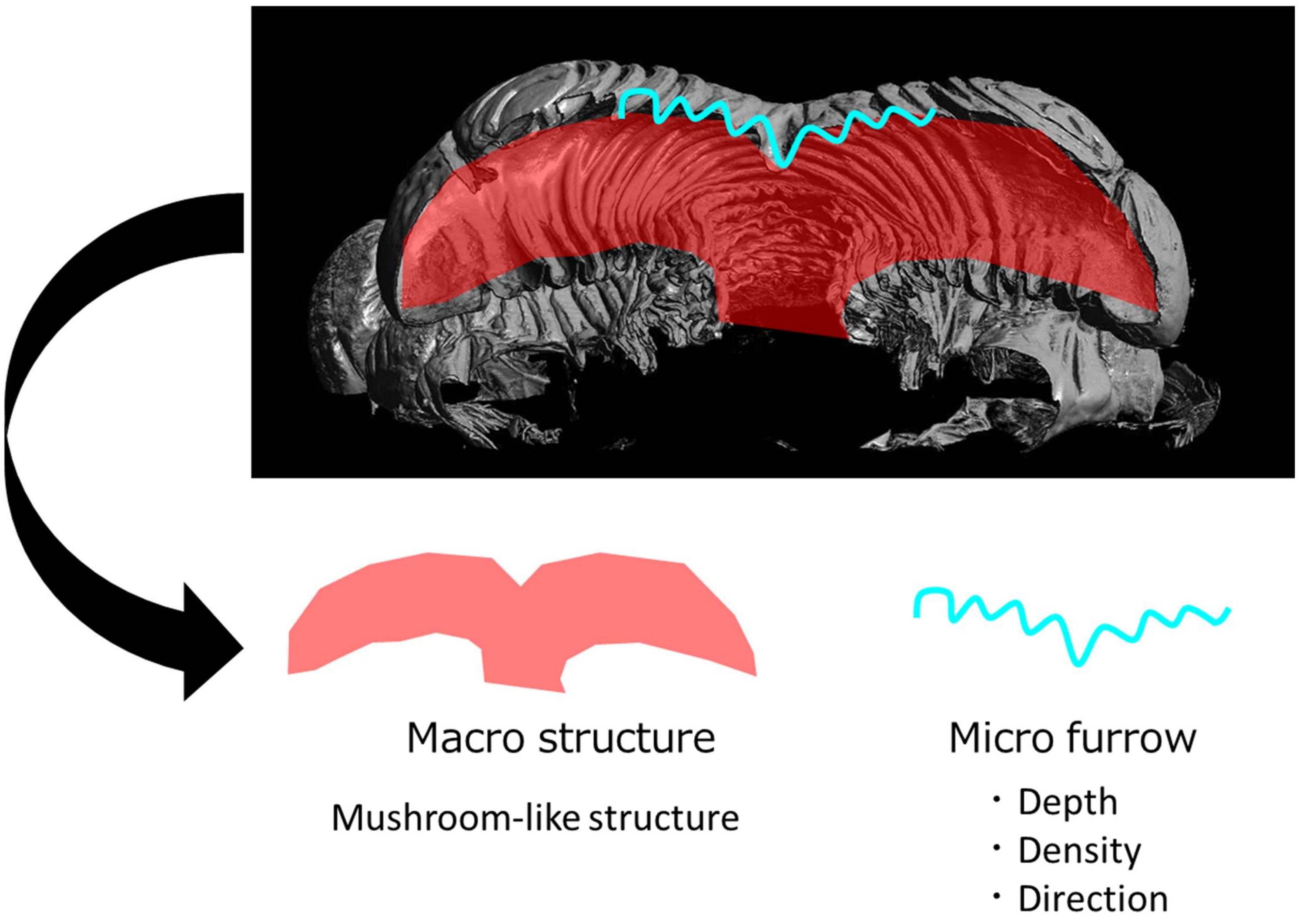

Figure S1. The morphology of the horn primordia

The horn primordium has a mushroom-like macro structure and surface micro furrows.

Micro furrows have depth, density and direction (2D furrow pattern).

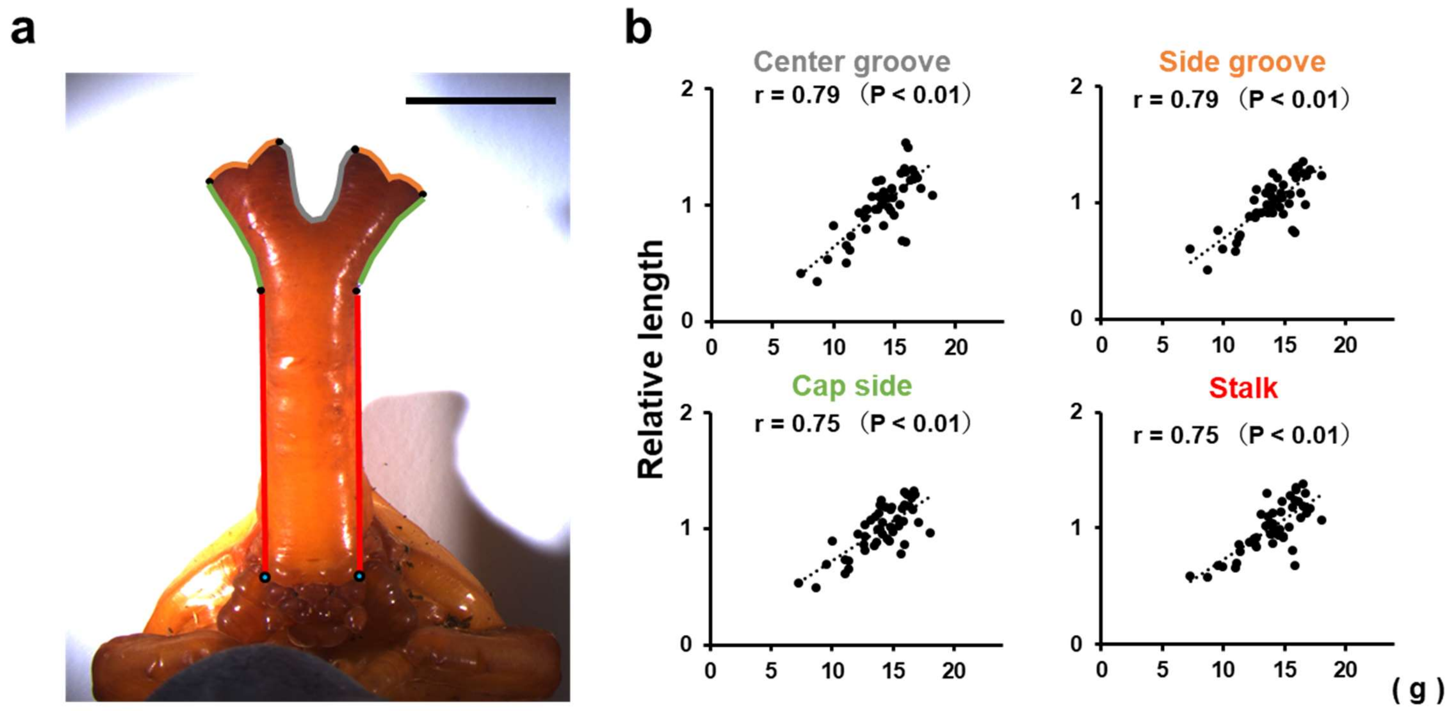

Figure S2. Relationship between body size and the morphology of the pupa

(a) Pupal horn with individual parts outlined. Landmarks were determined by inflection points. Scale bar indicates 10 mm. (b) Relationship between body size and the length of each part of the horn. Measured lengths of each horn part were strongly correlated with body size, and the scaling relationships (the slopes) with body size are nearly identical (n = 48).

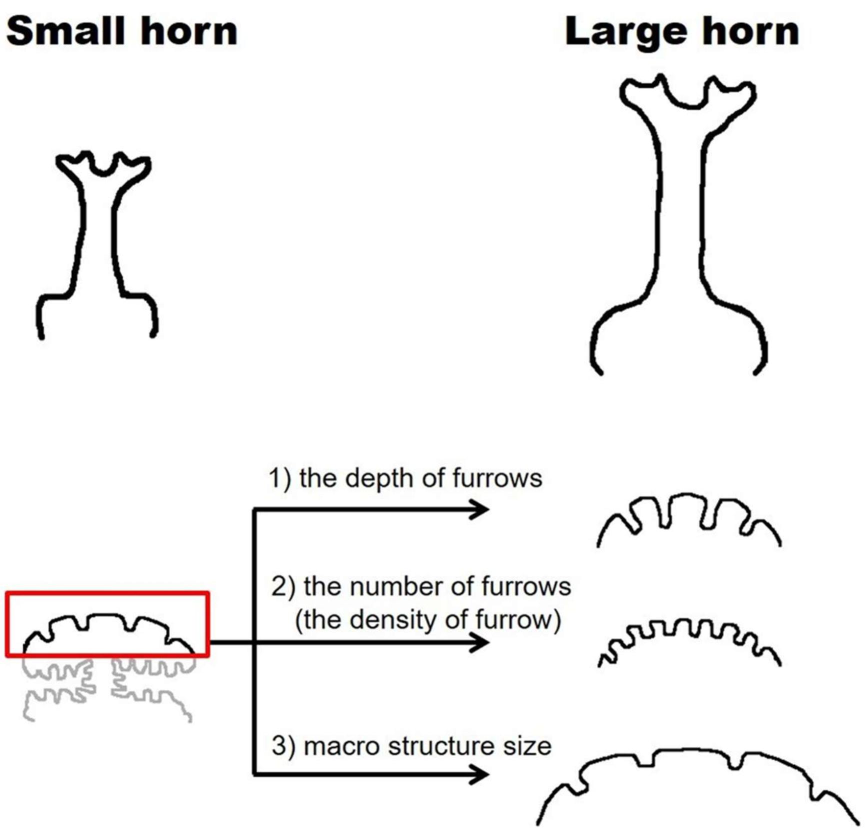

Figure S3. Three ways to increase the size of horn via changing morphological parameters of a primordium

There are three way to increase the size of horn via changing morphological parameters of a primordium. First is increasing the depth of the furrows. Second is increasing the number of furrows (in other words, increasing the density of the furrows). Third is increasing macro structure size.

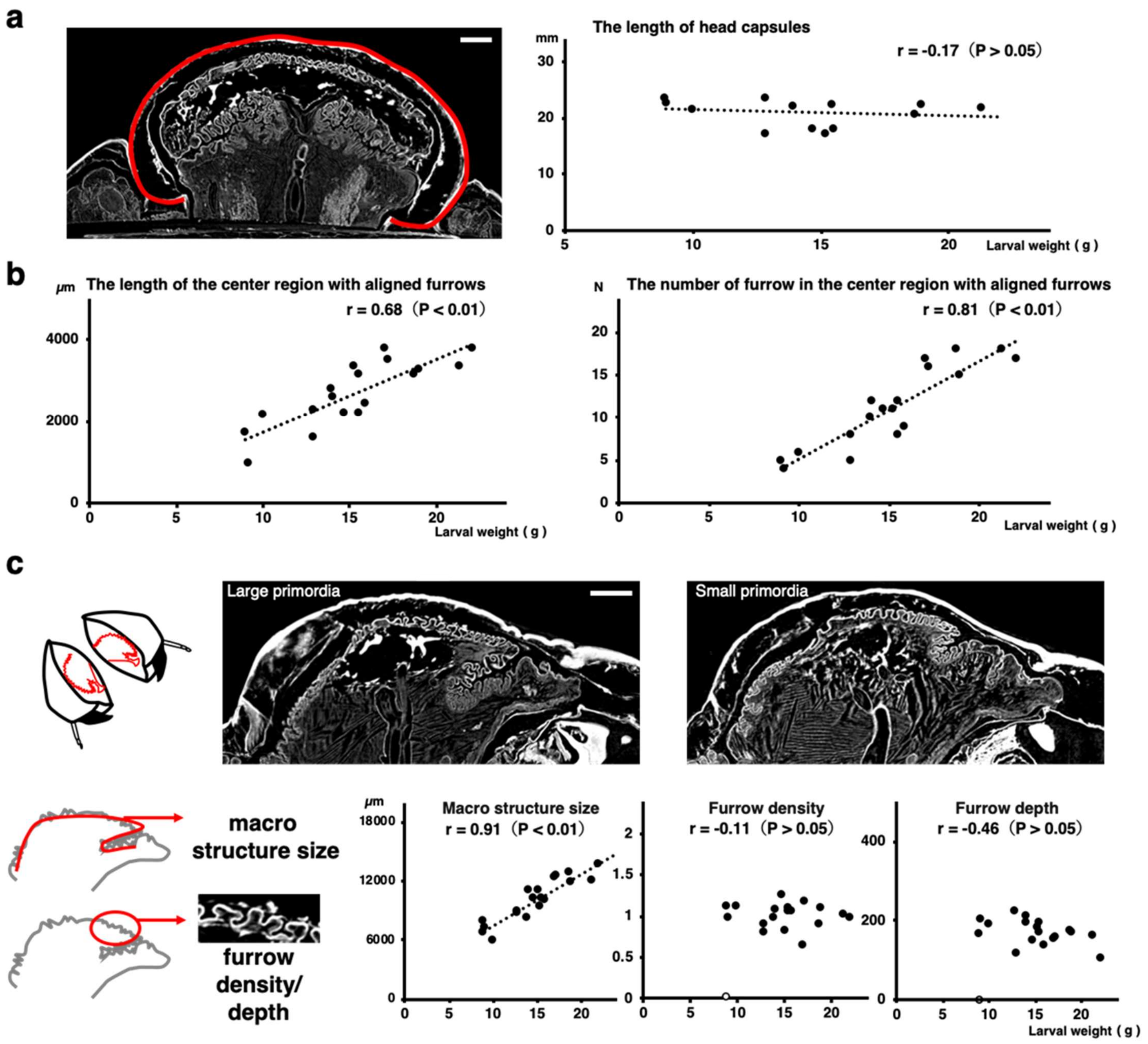

Figure S4. Relationship between body size and the morphology of the head capsules and primordia (sagittal section)

(a) Relationship between body size and the size of the head capsules. The correlation coefficients were not significant. ($n = 13$). Scale bar indicates 1 mm.

(b) Relationship between body size and the length of the center region with aligned furrows (left), and between body size and the number of furrows in the center region (right). From these data, we calculated the density of furrows (Fig. 1d). For center region length, the correlation coefficient was 0.68 ($p < 0.01$), and for furrow number, the correlation coefficient was 0.81 ($p < 0.01$) ($n = 19$).

(c) Relationship between body size and the primordial morphological parameters (macro structure size, density of the furrows, depth of the furrows) in the sagittal section. For macro structure size, the correlation coefficient was 0.91 ($p < 0.01$), for furrow density and depth, the correlation coefficients were not significant. In the smallest beetle (white marker: 8.95 g), obvious furrows could not be detected, so it was excluded from the analysis ($n = 19$). Scale bar indicates 1 mm.

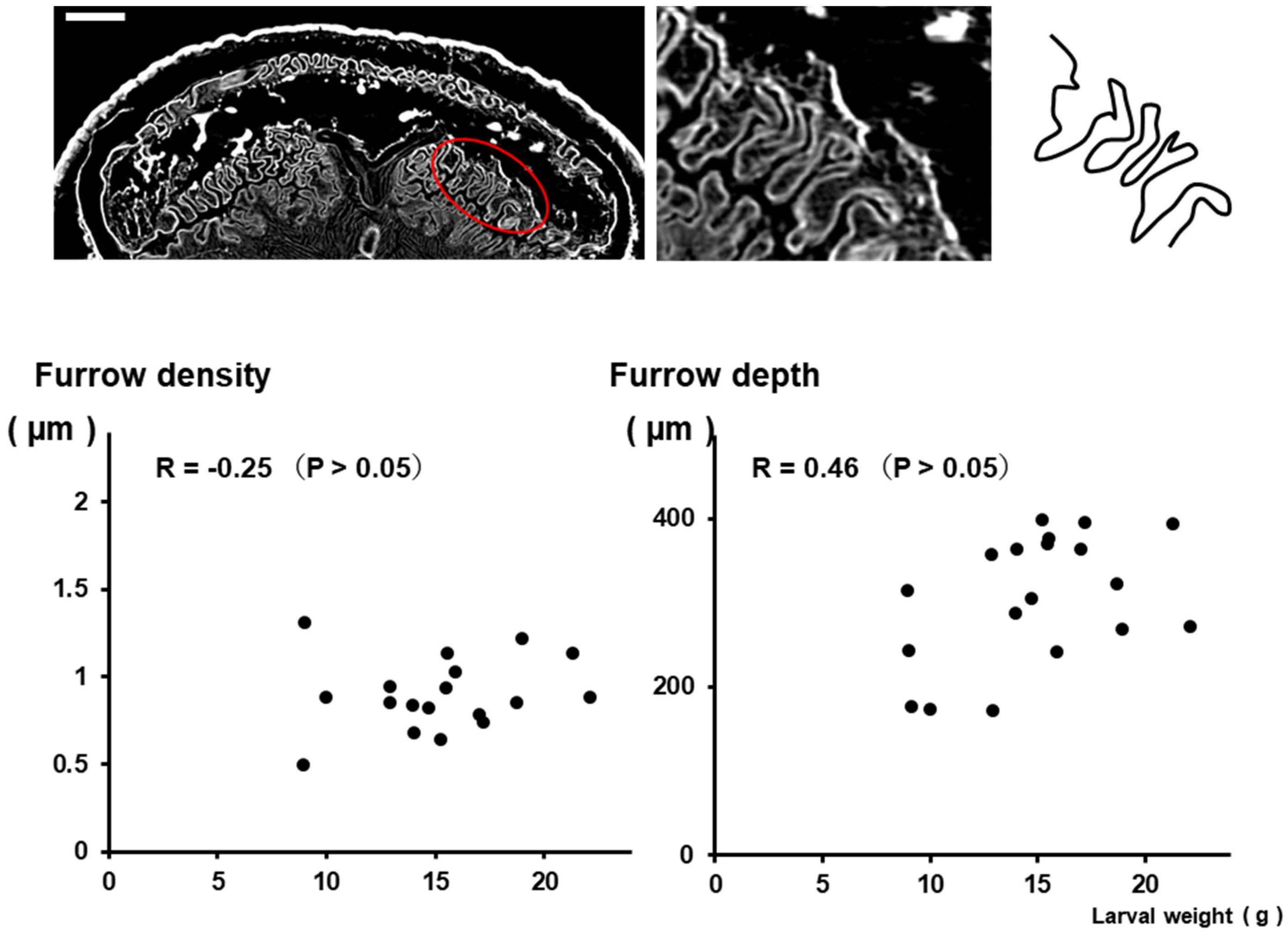

Figure S5. Relationship between body size and the morphology of the primordia (cap bottom region)

Relationship between body size and primordia parameters (macro structure size, density of the furrows and depth of the furrows) in the cap bottom region. For furrow density and depth, the correlation coefficients were not significant (n = 19). Scale bar indicates 1 mm.

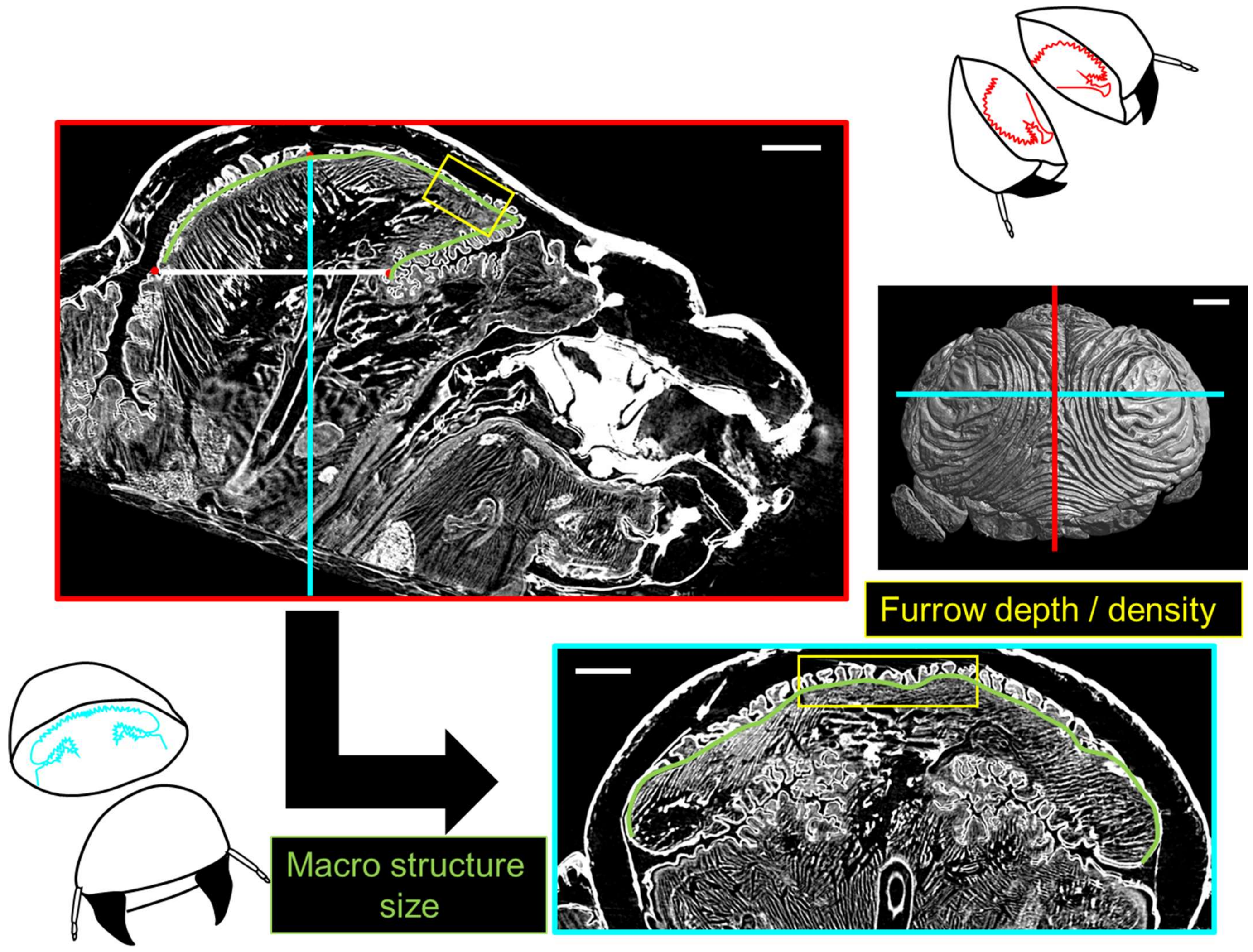

Figure S6. The area to analyze the morphological parameters

The equal-section images were obtained using landmarks such as the points of changing furrow direction and the inflection point of the macro structure of the primordia. Length of green line was measured as macro structure size. Furrow density and the depth were analyzed in the yellow region by measuring depth of each furrow and counting the number of furrows. Scale bar indicates 1 mm.

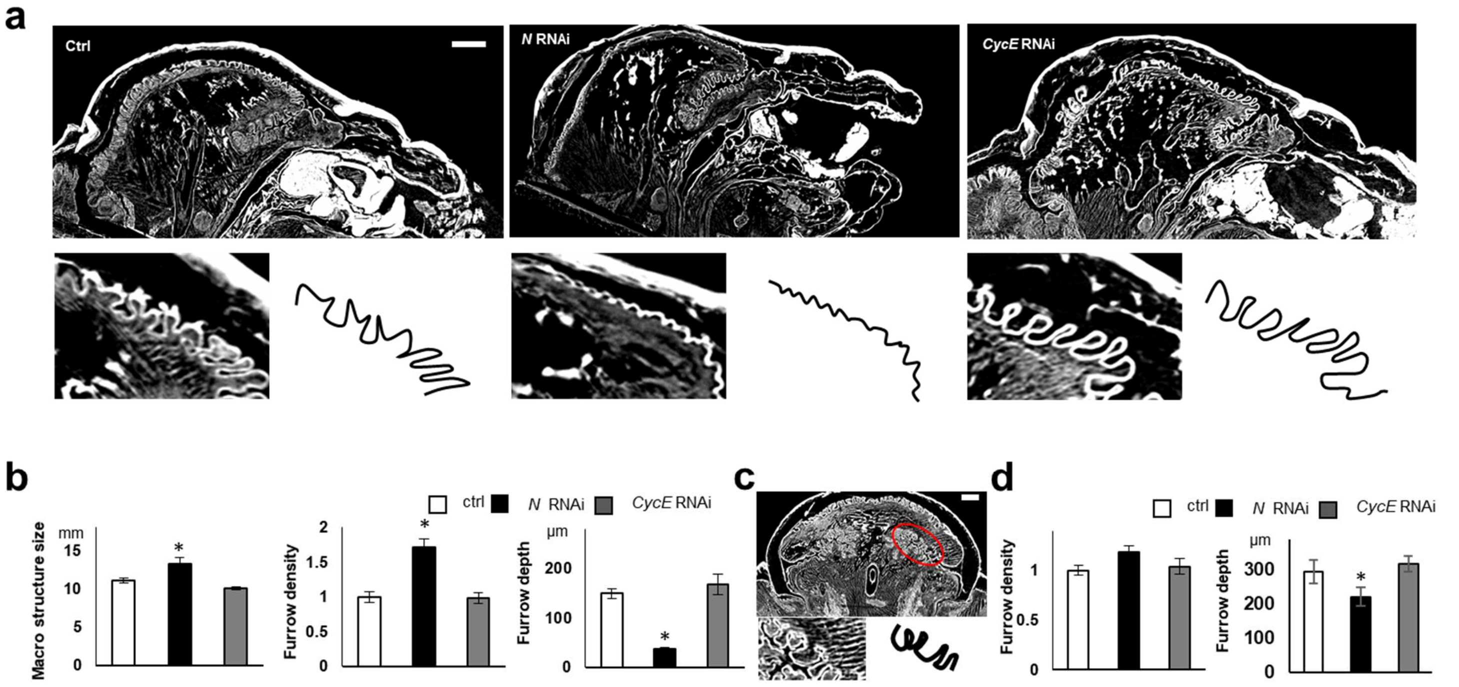

Figure S7. Morphological parameters of the primordia in *N* and *CycE* RNAi

(a) Comparison of sagittal section of the head just before pupation between control and *N* RNAi and *CycE* RNAi. (b) Quantitative data of macro structure size and furrow density and furrow depth from analysis (n = 7, 5, 6 for negative control, *N* RNAi, *CycE* RNAi, respectively). (c) Analysis point of the furrow in cap bottom region. (d) Quantitative data of furrow density and furrow depth in cap bottom region (n = 7, 5, 6 for negative control, *N* RNAi, *CycE* RNAi). Scale bar indicates 1 mm for (a) and (c).

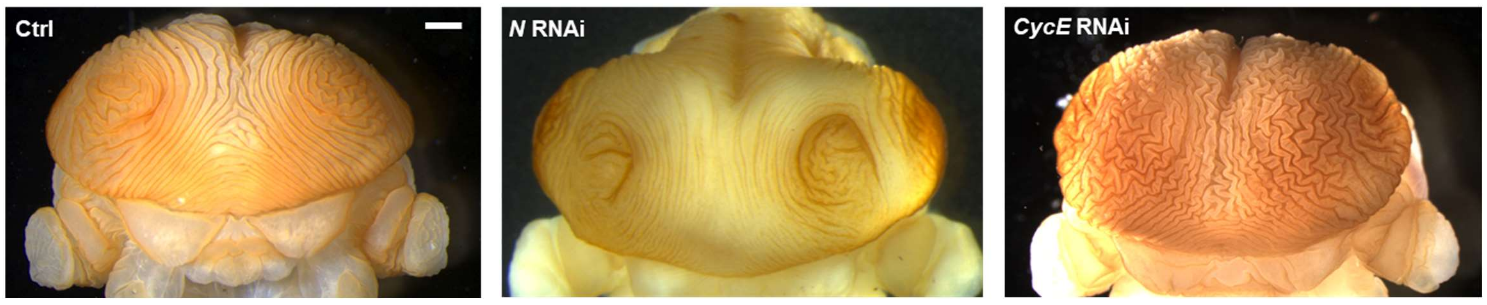

Figure S8. Comparison of 2D furrow pattern between control and *N* and *CycE* RNAi

Comparison of surface 2D furrow pattern between control and *N* and *CycE* RNAi. Two concentric-like furrows are observed in control and *N* RNAi. Zigzag pattern is observed in *CycE* RNAi.

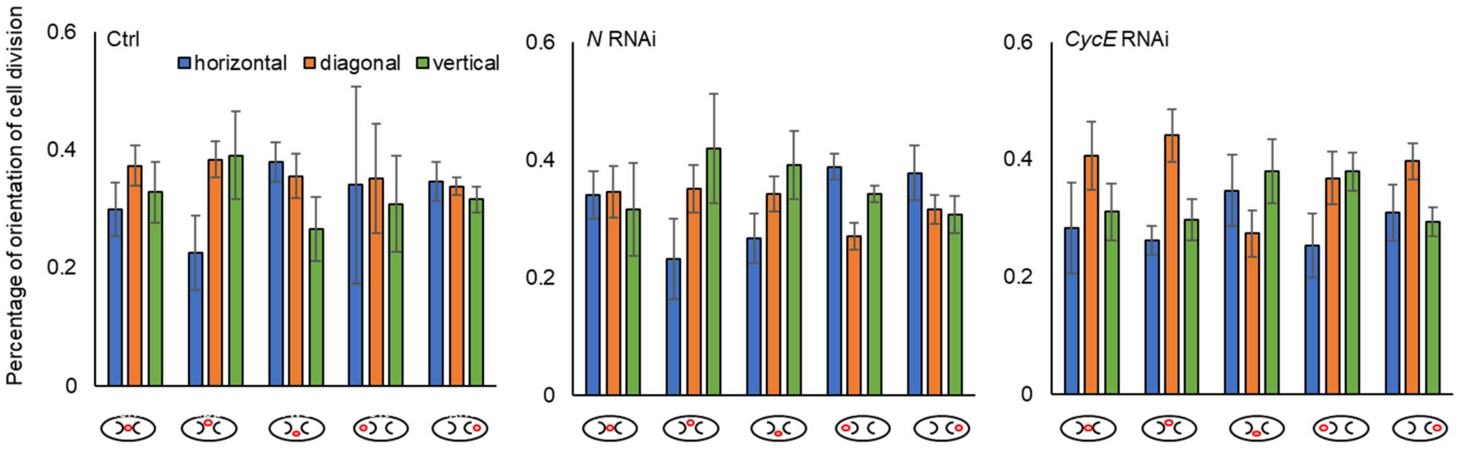

Figure S9. Comparison of cell division angle between control and *N* and *CycE* RNAi

Blue bars show the ratio of cells dividing in a horizontal direction (0 < θ ≤ 30). Orange bars show the ratio of cells dividing in a diagonal direction (30 < θ ≤ 60). Green bars show the ratio of cells dividing in a vertical direction (60 < θ ≤ 90). None of the RNAi treatments showed significant change of cell division orientation for any area.

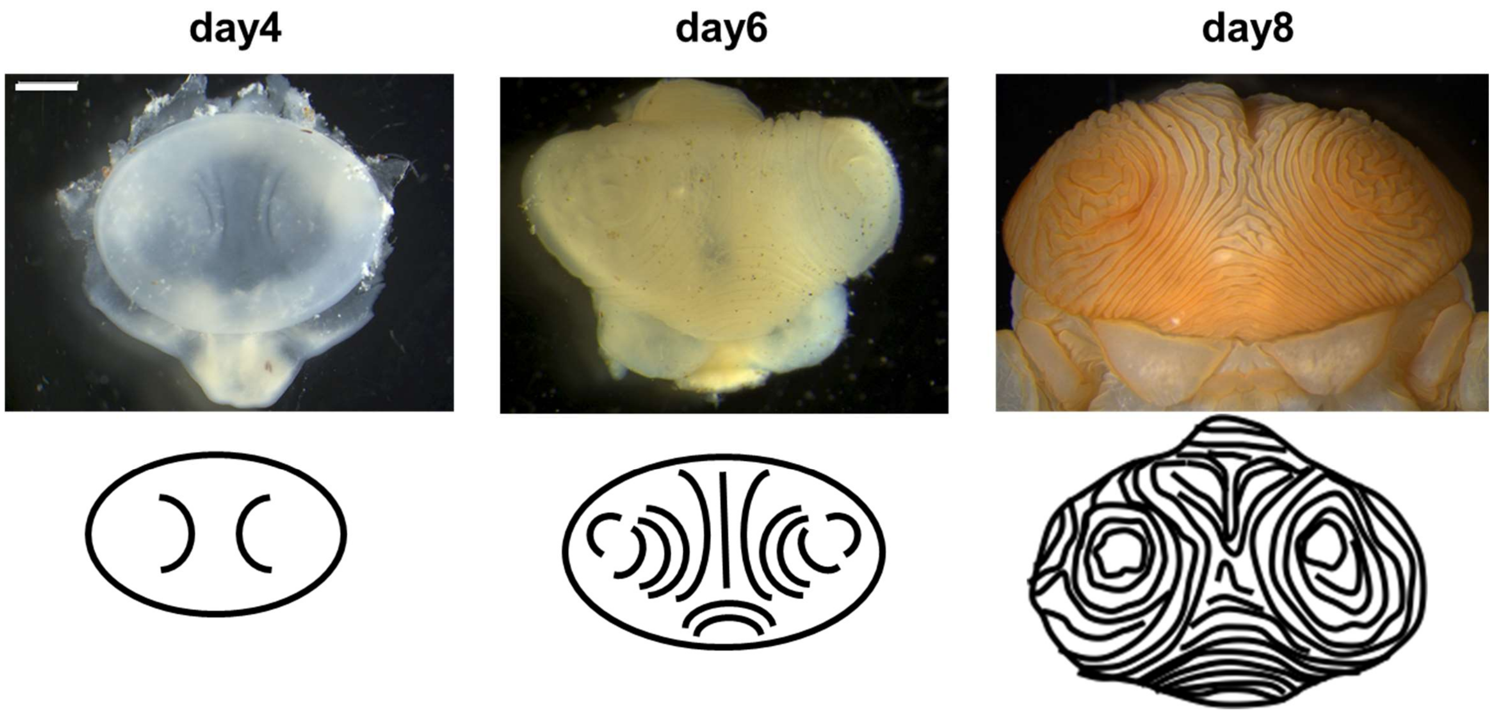

Figure S10. Chronological observation of the primordial furrow formation

A pair of crescent-shape furrows formed in the early stage. We used these furrows as a landmark to investigate cell division patterns across areas. Scale bar indicates 1 mm.

Gene name (accession number)	Forward sequence	Reverse sequence
N (IADJ01079691.1)	ACCGTTCAAACAAGGACTGG	CGTATCGCGAACTCTTGTGA
CycE (IADJ01090595.1)	CGTCGTAGAGAAATGCTGTAGC	GATCGGAGAAGGCGTCGACCTTTCGCG
mud (IADJ01075087.1)	TTGCCGGTCTTAATGTCCTC	GCCACTTTGCCGAGATGTAT
ds (IADJ01089570.1)	GTCAGGGAACTCGAGGTATCCGAAGG	CAGATCTAAATCCTTAGCCGGATGC
Optix (IADJ01054575.1)	ACGAAAAGCTCTCACGGAAA	ACTCGCTAATTGGGCTGCTA
rx (IADJ01019649.1)	AGTGAAACCGTTGAGGATGG	GAACTCTGACTTCGGGCAAG
egfp(control)	CCTGAAGTTCATCTGCACCA	TGCTCAGGTAGTGGTTGTCG

Table S1: List of primer used for gene amplification as dsRNA synthesis temperate
